# Raven’s Standard Progressive Matrices for Adolescents: A Case for a Shortened Version

**DOI:** 10.3390/jintelligence11040072

**Published:** 2023-04-13

**Authors:** Anne-Wil Kramer, Hilde M. Huizenga

**Affiliations:** Department of Developmental Psychology, University of Amsterdam, 1018 WS Amsterdam, The Netherlands

**Keywords:** Raven’s standard progressive matrices, short version, fatigue, motivation, adolescents

## Abstract

Cognitive ability of adolescents is often measured using the Raven’s Standard Progressive Matrices (RSPM). However, the RSPM knows a long administration time which may be suboptimal, as time-on-task effects are known to increase fatigue, to lower motivation, and to worsen performance on cognitive tasks. Therefore, a shortened version for adolescents was developed recently. In the current preregistered study we investigated this shortened version in a sample of adolescents (N = 99) of average educational backgrounds. We tested whether the shortened RSPM is a valid alternative to the original RSPM, which proved to be the case, as we observed a moderate to high correlation between the two versions. Moreover, we tested version effects on fatigue, motivation and performance. Fatigue was lower and motivation was higher after completing the short compared to the original version, and performance was better in the short compared to the original version. However, additional analyses suggested that beneficial version effects on performance were not due to reduced time-on-task, but due to the short version containing less difficult items than the original version. Moreover, version related differences in performance were not related to version related differences in fatigue and motivation. We conclude that the shortened version of the RSPM is a valid alternative to the original version, and that the shortened version is beneficial in terms of fatigue and motivation, but that these beneficial effects on fatigue and motivation do not carry over to performance.

## 1. Introduction

In educational and developmental studies, cognitive ability is often measured using the Raven’s Standard Progressive Matrices (RSPM; Raven 1989) (e.g., Cheung et al. 2016; Meinhardt-Injac et al. 2020). Since cognitive ability is often assessed not as a variable of interest, but rather as a background variable, administering the RSPM unnecessarily lengthens test batteries. That is, the RSPM knows a long administration time: it consists of 60 items taking up to 45 min to complete. This may be suboptimal, as time-on-task effects are known to increase fatigue, to lower motivation, and to worsen performance on cognitive tasks (for a review, see Müller and Apps 2019, see also Dekkers et al. 2017; Bioulac et al. 2012; Ackerman and Kanfer 2009; Boksem et al. 2005). Completing the full-length RSPM may thus leave participants fatigued and less motivated to subsequently complete other tasks or questionnaires. One solution may be to use a shorter version of the RSPM, such as a short 15-item version for adolescents that was recently developed by means of machine learning (Langener et al. 2022). In the current study, we tested whether this short version of the RSPM can be regarded as a valid alternative to the original version, and whether the short as compared to the original RSPM is beneficial in terms of fatigue, motivation, and performance.

To address these questions, we administered both the original and the short version of the RSPM to adolescents, and measured fatigue and motivation after each task-version. To assess validity, we determined the correlation between performance in the original and short version. To assess time-on-task effects, we tested whether fatigue was lower, motivation was higher, and performance was better in the short compared to the original version. In testing version effects on performance, we took care to rule out an alternative hypothesis, namely that performance is better in the short compared to the original version because the short version contains less difficult items. Finally, we tested whether version differences in performance were related to version differences in fatigue or in motivation.

## 2. Materials and Methods

This study was preregistered and can be found here: https://osf.io/phxrb. All materials and methods match the preregistration unless indicated otherwise.

### 2.1. Participants

A total of 99 adolescents aged between 13 and 16 (M_age_ = 14.52, SD_age_ = 0.63 years, N_female_ = 57) participated in this study. Participants were recruited via schools of average educational background (i.e., pre-vocational; more than 50% of Dutch high-school students attend this level). Three schools participated with a total of 5 classes. From all participants and parents, active consent was obtained. This study was approved by the ethical review board of the Psychology Department from the University of Amsterdam.

### 2.2. Procedure

Participation in the study involved two sessions: one session in which the original, and another session in which the short version of the RSPM was administered. There were four weeks in between sessions. The order of sessions was counterbalanced between classes. In each session, after completing the RSPM, participants indicated their current level of fatigue and motivation. Participants completed the tasks on a laptop or tablet in a classroom setting with 19 to 27 students present. Due to COVID-19 restrictions, researchers were not allowed to be physically present at schools. Therefore, during administration, a teacher was present in the classroom and a researcher was present online via videocall on a large screen. This way, the researcher could oversee the classroom and answer questions. All tasks and questions were filled out via Qualtrics (Qualtrics, Provo, UT, USA).

### 2.3. Materials

#### 2.3.1. Raven’s Standard Progressive Matrices

The original version of the RSPM consists of 60 items (Raven 1989). The task comprises a series of geometrical figures with a missing piece. Participants are instructed to select the missing piece among 6 to 8 alternatives. The RSPM consists of 5 item-sets that increase in difficulty both within- and between sets. The sets were administered according to their original sequence (i.e., starting with set A, ending with set E). The performance measure in the original version is defined as the percentage correct over all 60 items. That is, the sum score is divided by 60, then multiplied by 100.

The short version of the RSPM consists of 15 items selected from the original version. The items were selected using a machine learning approach (Langener et al. 2022). That is, the authors used regularized regression in combination with cross-validation to select a subset of items that could best predict the total score on the original version. The items were specifically selected in a sample of 13 to 16 year-olds. To obtain the performance measure in the short version, we calculated a predicted sum score based on the intercept and beta-weights derived from Langener et al. (2022). That is, we multiplied each item score (zero for incorrect, one for correct) with the associated beta-weight and added the intercept to derive a predicted sum score. We calculated percentage correct based on this predicted sum score. That is, the predicted sum score is divided by 60, then multiplied by 100.

#### 2.3.2. Fatigue and Motivation

In order to assess fatigue and motivation after completing each of the RSPM versions, participants answered a question about how fatigued they felt and how motivated they felt to continue working on school work. Both questions were answered on a 7-point Likert scale from 1 = “not at all”, to 7 = “very much”.

#### 2.3.3. Covariates Age and Sex

We may expect RSPM performance to increase with age. Therefore, we included age as linear covariate in our models. Sex differences may also occur in our variables of interest (e.g., Bugler et al. 2015), so we also included sex as a nominal covariate in our analyses.

#### 2.3.4. Analysis Plan

First, to assess validity, we calculated the Pearson correlation between performance in the original and the short version.

Then, we checked whether the order of testing (i.e., whether participants first performed the short or the original version) affected fatigue, motivation, and performance. Therefore, we fitted linear mixed models separately on fatigue, motivation, and performance, and examined fixed effects of task-order, task-version and their interaction. If task-order affected the outcome variable, we included task-order in the subsequent main analysis of interest.

To test the effect of time-on-task on fatigue, we fitted a linear mixed model on post-task fatigue scores. We examined fixed main effects of task-version, sex, and age. In addition, we examined two- and three-way interactions between task-version, sex and age. We fitted the same model on post-task motivation scores and on RSPM performance scores.

Finally, we tested with a regression analysis whether version related differences in performance were associated with version related differences in fatigue and motivation. This analysis was not preregistered, and thus exploratory. In all linear mixed models, we standardized continuous predictor variables and we contrasted categorical predictor variables (i.e., task-version: short = −1, long = 1; task-order: short first = −1, long first = 1; and sex: male = −1, female = 1). Additionally, in all linear mixed models, the intercept was allowed to vary over participants in order to take into account the repeated nature of the data. For these analyses, we used the lme4 package (Bates et al. 2015) in R version 4.1.3.

## 3. Results

### 3.1. Descriptives

Table 1 shows descriptives of fatigue, motivation and performance in both the short and original version of the RSPM. In addition, we calculated reliability estimates together with their 95% credibility intervals (CI). We did so in a Bayesian framework, taking into account the impact of sampling error (Pfadt et al. 2022). Cronbach’s α was 0.61 (95% CI [0.51, 0.72]) for the short version and 0.86 (95% CI [0.82, 0.90]) for the original version.^1^

### 3.2. Correlation between Original and Short Version of the RSPM

We found a moderately high correlation between performance in the original and the short version (Pearson’s r = 0.62, *p* < .001). This indicates that the 15-item version serves as valid alternative to the original version.

### 3.3. Checks on Task Order Effects

Linear mixed modeling showed no main effects of task-order on fatigue (b = −0.17, SE = 0.15, 95% CI [−0.47, 0.13], t = −1.14, *p* = .257), motivation (b = 0.06, SE = 0.15, 95% CI [−0.24, 0.36], t = 0.42, *p* = .677) or performance (b = −0.05, SE = 0.74, 95% CI [−1.51, 1.41], t = −0.07, *p* = .622). Importantly, we found no interaction between task-order and task-version on motivation (b = 0.06, SE = 0.07, 95% CI [−0.09, 0.20], t = 0.81, *p* = .420) and performance (b = 0.51, SE = 0.51, 95% CI [−0.50, 1.51], t = 0.99, *p* = .091), indicating that motivation and performance differences related to task-version did not depend on what version was performed first. However, we found an interaction between task-order and task-version on fatigue (b = −0.31, SE = 0.10, 95% CI [−0.51, −0.10], t = −2.92, *p* = .004). Follow-up regressions indicated that when participants started with the short version, they reported more fatigue after completing the original version (b = 0.88, SE = 0.17, 95% CI [0.54, 1.22], t = 5.20, *p* < .001) compared to when they started with the original version (b = 0.28, SE = 0.18, 95% CI [−0.09, 0.63], t = 1.49, *p* = 0.140). Therefore, we included task-order in the remaining analyses on fatigue but not on motivation and performance.

### 3.4. Time-on-Task Effects on Fatigue, Motivation and Performance

Linear mixed modeling on fatigue showed that fatigue was lower after completing the short compared to the original version (main effect task-version: b = 0.57, SE = 0.10, 95% CI [0.36, 0.77], t = 5.42, *p* < .001). We found no main effects of age, sex nor any interactions. In addition, we found the aforementioned interaction between task-version and task-order (interaction: b = −0.27, SE = 0.11, 95% CI [−0.48, −0.06], t = −2.56, *p* = .012). No other interactions were found (see Supplementary Online Material; SOM Table S2).

Next, linear mixed modeling on motivation showed that motivation was higher after completing the short compared to the original version (main effect task-version: b = −0.21, SE = 0.07, 95% CI [−0.36, −0.07], t = −2.92, *p* = .004). We found no main effects of age or sex, nor any interactions (SOM Table S2).

Finally, linear mixed modeling on performance showed that performance was better in the short compared to the original version (main effect task-version: b = −1.98, SE = 0.51, 95% CI [−2.98, −0.97], t = −3.89, *p* < .001). No other main or interaction effects were found (SOM Table S2).

**Figure 1:**
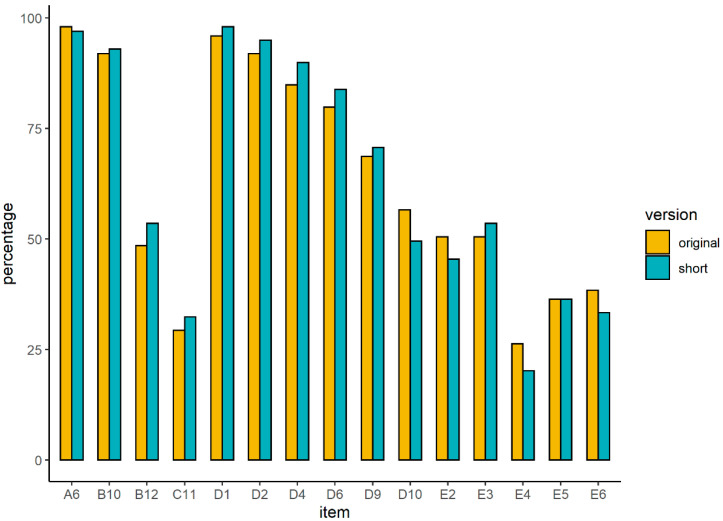
Percentage correct on original and short version items. Note: We only depicted items from the original version that were also administered in the short version. We tested per item whether item-performance differed between versions using a series of logistic regressions. Results indicated that performance on all items was similar across versions (SOM Table S6).

Exploratorily (i.e., not preregistered), to rule out the alternative explanation that performance was better in the short version because it contained less difficult items, we also calculated a weighted 15-item score for the original version (similar to the performance measure used for the short version). We ran the same performance model again while now comparing performance on the same set of (weighted) 15-items in both versions. The effect of task-version now became nonsignificant (b = −0.08, SE = 0.18, 95% CI [−0.43, −0.27], t = −0.43, *p* = .668) (see also Figure 1), as well as all other main and interaction effects (SOM Table S5). This suggests that better performance in the short version is not due to time on task effects, but due to the short version containing less difficult items.

### 3.5. Fatigue and Motivation Differences Do Not Predict Performance Differences

We found no relation between version differences in performance on the one hand and version differences in fatigue (b = −0.28, SE = 1.08, 95% CI [−2.43, 1.86], t = −0.26, *p* = .793) and motivation (b = −0.51, SE = 1.08, 95% CI [−2.67, 1.64], t = −0.47, *p* = .638) on the other (SOM Table S7). This indicates that performance differences between versions are not driven by either fatigue differences nor motivation differences.

## 4. Discussion

The current study examined the properties of a recently developed short version of the Raven Standard Progressive Matrices (RSPM) in an adolescent sample. Performance in the short version showed a moderately high correlation with performance in the original version, suggesting that the shortened version is a valid alternative to the original version. In line with our hypotheses, results showed that fatigue was lower and motivation was higher after completing the short as compared to the original RSPM. In addition, performance was better in the short compared to the original version. However, better performance on the short version was likely not due to time-on-task effects, as version related differences disappeared when comparing performance scores based on the same set of items in the original and short version. Moreover, version related differences in motivation or fatigue were not predictive of version related differences in performance.

Our results indicate that the short version serves as valid alternative to the original version for adolescents as evidenced by the relation between the two versions. This study adds in two ways to previous studies investigating validity of short versions of the (advanced) RPM (e.g., Arthur et al. 1999; Myszkowski and Storme 2018). First, participants in our study completed both the short and original version, which allowed us to test the relation between performance on the two versions. Second, our adolescent sample comes from average educational backgrounds. This is important, because, as noted by others, the RSPM is less appropriate for individuals with high expected levels of intelligence, as evidenced by the regularly observed ceiling-effects (in adults) (e.g., Myszkowski and Storme 2018). In our sample, only three percent of participants achieved a perfect score on the short version, supporting the notion that the short RSPM serves as valid alternative to the original version for the average adolescent.

Besides reducing test-length, another advantage of using the short over the original version is that after completing the short version, adolescents suffered less from fatigue and were more motivated to complete other tasks. This result is in line with earlier studies reporting similar time-on-task effects on fatigue (Ackerman and Kanfer 2009; Müller and Apps 2019). However, those studies have not assessed participants’ motivation to continue working on other tasks. Demonstrating these effects in the short RSPM is important, as the RSPM is often administered as part of a larger test-battery. Administering the original version could therefore adversely affect scores on subsequent tests; something that can be overcome by administering the short version.

Notably, these time-on-task effects on fatigue and motivation do not carry over to performance; fatigue and motivation differences did not predict the observed performance difference between versions. This is in line with earlier research reporting that time-on-task increased fatigue, but fatigue in turn did not adversely affect performance on cognitive tasks (Ackerman and Kanfer 2009; Ackerman et al. 2010). The authors argue that experiencing fatigue reflects a functional process that is associated with increased levels of exerted effort and may therefore even be associated with better performance on the task that fatigued them. Yet, fatigue will still affect (cognitive) performance on subsequent tasks. Thus, when administering long test-batteries in adolescent samples, it would still be advised to use the short RSPM version.

The current study also knowns some limitations. First, we examined adolescents from average educational backgrounds. While this group comprises the largest group of students in the Dutch school system, we do not know whether similar effects would be observed in a broader sample of adolescents of more varied educational backgrounds. Thus, future research may replicate the current study in a more diverse sample. Second, we only measured fatigue and motivation after RSPM completion. It could be argued that the post- minus pre-task decline in fatigue is better predictive of RSPM performance. Others have also reported that pre-task fatigue is a predictor of post-task fatigue (Ackerman and Kanfer 2009). Thus, future research may consider to measure fatigue and motivation also pre-task. Third, it may be argued that our conclusion that there is no time-on-task effect on performance in the entire task should be qualified, as there still may exist time-on-task effects at the end of the task. However, inspection of the results in Figure 1 do not support this argument: Performance on items at the end of the task also does not differ between original and short versions. Finally, to reduce burden on participants we have used single-item measures to assess fatigue and motivation. However, single-item instruments may be less reliable than multiple item instruments. Therefore, future studies are advised to use a more comprehensive assessment, such as the NASA-TLX (Hart and Staveland 1988).

The current study has several implications for the use of the RSPM in developmental and educational studies. First, as the shortened version is a valid alternative to the original RSPM for adolescents, the shortened version may be used as a screener for cognitive performance in these studies. This leaves more time for investigation of the key variables of interest. Second, as the performance score of the short version was higher than the performance score of the original version, we do not advise to compare performance in the short version with norm tables developed for the original version, as this may lead to overestimation of IQ scores. Rather, we advise on using the raw short version performance score.

## 5. Conclusions

Together, we conclude that the shortened version of the RSPM is a valid alternative to the original version, and that the shortened version is beneficial in terms of fatigue and motivation, but that these beneficial effects on fatigue and motivation do not carry over to performance. While this study calls for further investigations in other populations, we suggest using the short version of the RSPM in adolescent populations that share similarities with our sample when time is scarce.

## Figures and Tables

**Table 1:** Means and standard deviations (between brackets) of fatigue, motivation and performance in the short and original versions of the RSPM.

	Short Version	Original Version
Fatigue	3.38 (1.73)	4.43 (1.94)
Motivation	3.77 (1.67)	3.36 (1.70)
Performance	71.84 (3.94)	67.92 (11.86)

## Data Availability

Data are openly available and can be found here https://osf.io/d5jph/.

## Note

1 We also calculated other reliability indices. For the short version, McDonalds ω = 0.54, 95% CI [.40, .67], Guttman’s λ^2^ = 0.65, 95% CI [0.56, 0.75], Guttman’s λ^6^ = 0.75, 95% CI [0.69, 0.82], GLB = 0.83, 95% CI [0.78, 0.88]. For the original version, McDonalds ω = 0.83, 95% CI [0.79, 0.87], Guttman’s λ^2^ = 0.87, 95% CI [0.84, 0.90], Guttman’s λ^6^ = 0.97, 95% CI [0.97, 0.98], GLB = 0.99, 95% CI [0.98, 0.99].

## Supplementary Materials

The following supporting information can be downloaded at: https://www.mdpi.com/article/10.3390/jintelligence11040072/s1, Figure S1: Plotted residuals (left column) and Q-Q normal plots (right column) for the models fit on fatigue, motivation and performance; Figure S2: Percentage correct for how often each item was answered correctly in the original version; Table S1: Results from linear mixed models on effects of task order and task version on fatigue, motivation, and performance (manipulation checks; Table S2: Results from linear mixed models on fatigue, motivation and performance (main analyses); Table S3: Means and standard deviations (between brackets) for the different percentage correct scores on the original and short versions; Table S4: Correlations between the various performance measures; Table S5: Results from linear mixed models on performance (now using the weighted 15-item measure for the original version); Table S6: Results from a series of linear mixed logistic regressions testing the effect of version on performance per item; Table S7: Results from linear model testing whether fatigue and motivation differences predict performance differences. Reference (Schielzeth et al. 2020) is cited in the Supplementary Material.
