# Supplementary material for: Raven’s Standard Progressive Matrices for Adolescents: A Case for a Shortened Version
Source: J Intell. 2023 Apr 13;11(4):72. doi: 10.3390/jintelligence11040072 (PMC10144826; doi:10.3390/jintelligence11040072)
Supplement: Supplementary file 1 [file jintelligence-11-00072-s001.zip › jintelligence-2251912-supplementary.pdf]

Supplementary Online Materials (SOM)

Results preregistered linear mixed models

For our linear mixed models, the assumption of linearity was met for the models fit on fatigue, motivation and performance (see Figure S2 for plotted residuals). Although violating the assumptions of homoscedasticity and normality is not problematic for robust estimation in linear mixed models (Schielzeth et al., 2020), we did evaluate these two assumptions. The assumption of homoscedasticity was assessed with Levene's tests, and was met for the models fit on fatigue, $F(1, 198) = 0.73$, $p = .39$, and motivation, $F(1, 198) = 0.02$, $p = .90$, but not performance, $F(1, 198) = 14.57$, $p < .001$ (variance was higher for the original than for the short version, see SOM Figure S2).

The assumption of normality was tested with Kolmogorov-Smirnoff tests, and was met for the model fit on fatigue for the short ($D = 0.12$, $p = .14$) but not original version ($D = 0.16$, $p = .01$), was met for the model fit on motivation for both the short ($D = 0.12$, $p = .21$) and original ($D = 0.08$, $p = .56$) version, and was not met for the model fit on performance for the short ($D = 0.21$, $p < .001$) nor the original version ($D = 0.43$, $p < .001$).

Figure S1

Plotted residuals (left column) and Q-Q normal plots (right column) for the models fit on fatigue, motivation and performance.

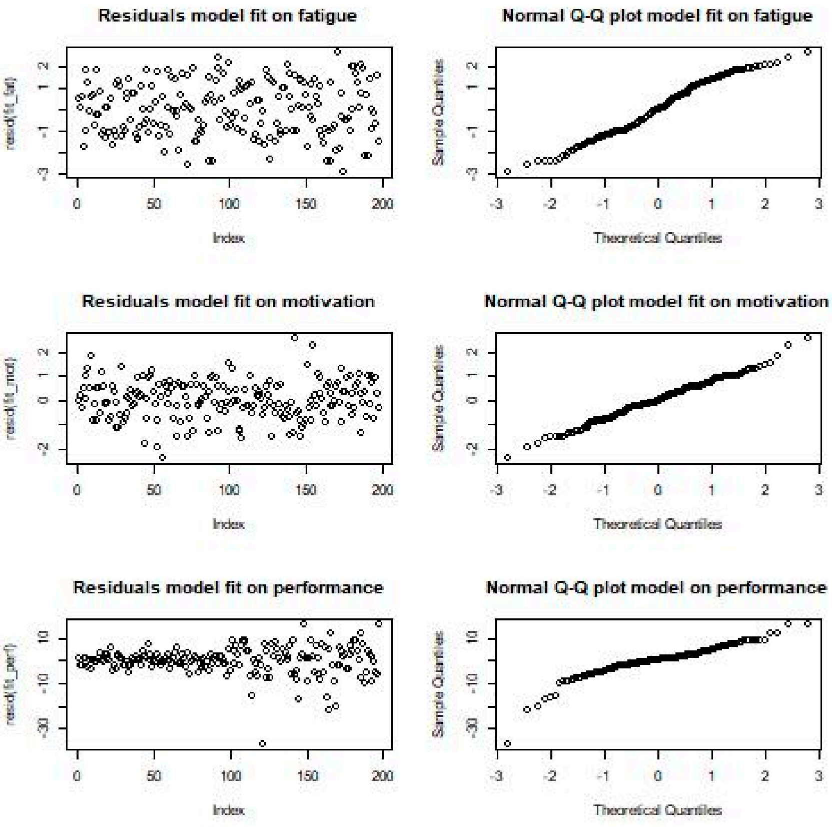

Note: With the plots in the left hand column we check whether residual variance is similar across versions. There were 99 participants, the first 99 indices represent participant IDs in the short version, while the second 99 indices represent participant IDs in the original version. The y-axes represent residual variance.

Table S1

Results from linear mixed models on effects of task order and task version on fatigue, motivation, and performance (manipulation checks).

			Fatigue		
Predictors	*b*	*SE*	95% CI	*t*	*p*
Intercept	3.95	0.15	3.65 – 4.24	26.16	**<.001**
task order	-0.17	0.15	-0.47 – 0.13	-1.14	.257
task version	0.58	0.10	0.37 – 0.78	5.53	**<.001**
task order * task version	-0.31	0.10	-0.51 - -0.10	-2.92	**.004**
Random effects					
σ^2	2.10	1.45			
Random intercept	1.13	1.06			
ICC	0.35				
Marginal R^2 / conditional R^2	0.11 / 0.42				
			Motivation		
Predictors	*b*	*SE*	95% CI	*t*	*p*
Intercept	3.54	0.15	3.24 – 3.84	23.27	**<.001**
task order	0.06	0.15	-0.24 – 0.36	0.42	.677
task version	-0.23	0.07	-0.37 - -0.08	-3.07	**.003**
task order * task version	0.06	0.07	-0.09 – 0.20	0.81	.420
Random effects					
σ^2	1.05	1.02			
Random intercept	1.69	1.30			
ICC	0.62				
Marginal R^2 / conditional R^2	0.02 / 0.62				
			Performance		
Predictors	*b*	*SE*	95% CI	*t*	*p*
Intercept	69.91	0.74	68.45 – 71.37	94.35	**<.001**
task order	-0.05	0.74	-1.51 – 1.41	-0.07	.95
task version	-2.10	0.51	-3.11 – 1.09	-4.11	**<.001**
task order * task version	0.51	0.51	-0.50 – 1.51	0.99	.326
Random effects					
σ^2	50.18	7.08			
Random intercept	27.24	5.22			
ICC	0.35				
Marginal R^2 / conditional R^2	0.05 / 0.39				

Note: Marginal R^2 denotes variance explained by fixed factors while conditional R^2 denotes variance explained by both fixed and random factors.

Table S2

Results from linear mixed models on fatigue, motivation and performance (main analyses).

			Fatigue		
Predictors	*b*	*SE*	95% CI	*t*	*p*
Intercept	3.94	0.15	3.64 – 4.23	26.04	**<.001**
task order	-0.13	0.15	-0.43 – 0.17	-0.84	.405
task version	0.57	0.10	0.36 – 0.77	5.42	**<.001**
age	0.25	0.15	-0.05 – 0.55	1.65	.103
sex	-0.11	0.14	-0.38 – 0.16	-0.79	.432
task order * task version	-0.27	0.11	-0.48 - -0.06	-2.56	**.012**
age * task version	0.15	0.11	-0.06 – 0.36	1.37	.174
sex * task version	-0.01	0.10	-0.20 – 0.18	-0.10	.921
age * sex	0.02	0.15	-0.27 – 0.31	0.15	.882
age * sex * task version	-0.08	0.10	-0.27 – 0.12	-0.74	.462
Random effects					
σ^2	2.11	1.45			
Random intercept	1.12	1.06			
ICC	0.35				
Marginal R^2 / conditional R^2	0.14 / 0.44				
			Motivation		
Predictors	*b*	*SE*	95% CI	*t*	*p*
Intercept	3.55	0.15	3.25 – 3.85	23.40	**<.001**
task version	-0.21	0.07	-0.36 - -0.07	-2.92	**.004**
age	-0.16	0.15	-0.46 – 0.14	-1.04	.301
sex	0.07	0.14	-0.20 – 0.35	0.52	.607
age * task version	-0.07	0.07	-0.22 – 0.08	-0.97	.335
sex * task version	0.06	0.07	-0.07 – 0.20	0.91	.368
age * sex	-0.02	0.15	-0.31 – 0.27	-0.14	.886
age * sex * task version	0.00	0.07	-0.14 – 0.15	0.06	.953
Random effects					
σ^2	1.06	1.03			
Random intercept	1.70	1.30			
ICC	0.62				

Marginal R^2 / conditional R^2	0.03 / 0.63				
Performance (total score original version and weighted 15-item score short version)					
Predictors	*b*	*SE*	95% CI	*t*	*p*
Intercept	69.97	0.73	68.53 – 71.41	95.79	**<.001**
task version	-1.98	0.51	-2.98 – -0.97	-3.89	**<.001**
age	-0.87	0.74	-2.32 – 0.59	-1.17	.245
sex	0.65	0.68	-0.68 – 1.99	0.97	.337
age * task version	-0.18	0.52	-1.21 – 0.84	-0.36	.723
sex * task version	0.51	0.47	-0.42 – 1.44	1.07	.285
age * sex	0.46	0.46	-0.94 – 1.85	0.64	.521
age * sex * task version	0.41	0.50	-0.57 – 1.39	0.83	.412
Random effects					
σ^2	50.66	7.12			
Random intercept	26.28	5.13			
ICC	0.34				
Marginal R^2 / conditional R^2	0.08 / 0.39				
Performance (total score original version and weighted 15-item score short version): now also including fatigue and motivation as predictors					
Predictors	*b*	*SE*	95% CI	*t*	*p*
intercept	69.80	0.75	68.32 – 71.28	93.06	**<.001**
task version	-2.24	0.56	-3.34 – -1.14	-4.00	**<.001**
fatigue	0.92	0.69	-0.45 – 2.29	1.32	.187
motivation	-0.45	0.72	-1.87 – 0.97	-0.62	.537
age	-1.17	0.75	-2.65 – 0.30	-1.57	.119
sex	0.69	0.68	-0.65 – 2.03	1.02	.311
fatigue * task version	0.77	0.63	-0.46 – 2.01	1.23	.219
motivation * task version	-0.49	0.61	-1.70 – 0.72	-0.80	.423
fatigue * motivation	0.45	0.65	-0.84 – 1.74	0.69	.494
age * task version	-0.50	0.55	-1.58 – 0.58	-0.91	.366
sex * task version	0.56	0.49	-0.40 – 1.53	1.15	.483
age * sex	0.50	0.70	-0.89 – 1.88	0.71	.255
fatigue * motivation * task version	0.27	0.58	-0.87 – 1.41	0.47	.640
age * sex * task version	0.46	0.50	-0.54 – 1.45	0.90	.370
Random effects					
σ^2	52.34	7.24			
Random intercept	24.34	4.93			
ICC	0.32				
Marginal R^2 / conditional R^2	0.10 / 0.38				

Table S3

Means and standard deviations (between brackets) for the different percentage correct scores on the original and short versions.

Based on	Percentage correct
1. Total score (original version)	67.92 (11.86)
2. Weighted 15-item score (original version)	71.82 (4.37)
3. Weighted 15-item score (short version)	71.84 (3.94)

Table S4

Correlations between the various performance measures.

	1	2	3
1. Total score (original version)	-		
2. Weighted 15-item score (original version)	.84	-	
3. Weighted 15-item score (short version)	.62	.62	-

Note: all *p's* < .001.

Table S5

Results from linear mixed models on performance (now using the weighted 15-item measure for the original version).

Performance (weighted 15-item score original version and weighted 15-item score short version)					
Predictors	*b*	*SE*	95% CI	*t*	*p*
Intercept	71.89	0.36	71.17 – 72.61	197.08	**<.001**
task version	-0.08	0.18	-0.43 – 0.27	-0.43	.668
age	-0.44	0.37	-1.17 – 0.29	-1.19	.237
sex	0.02	0.34	-0.65 – 0.68	0.05	.959
age * task version	0.24	0.18	-0.11 – 0.60	1.34	.182
sex * task version	-0.15	0.16	-0.47 – 0.18	-0.89	.374
age * sex	0.16	0.35	-0.54 – 0.85	0.44	.658
age * sex * task version	0.11	0.11	-0.23 – 0.45	0.64	.525
Random effects					
σ^2	6.13	2.48			
Random intercept	9.77	3.13			
ICC	0.61				
Marginal R^2 / conditional R^2	0.02 / 0.62				

Performance (weighted 15-item score original version and weighted 15-item score short version): now also including fatigue and motivation as predictors					
Predictors	*b*	*SE*	95% CI	*t*	*p*
intercept	71.93	0.37	71.20 – 72.67	192.83	**<.001**
task version	-0.09	0.20	-0.49 – 0.30	-0.47	.640
fatigue	0.12	0.28	-0.44 – 0.68	0.43	.668
motivation	-0.11	0.31	-0.72 – 0.51	-0.34	.736
age	-0.47	0.37	-1.20 – 0.27	-1.25	.214
sex	0.03	0.34	-0.65 – 0.70	0.07	.943
fatigue * task version	-0.20	0.23	-0.66 – 0.26	-0.87	.383
motivation * task version	-0.18	0.23	-0.63 – 0.26	-0.81	.418
fatigue * motivation	0.07	0.27	-0.47 – 0.60	0.25	.806
age * task version	0.23	0.19	-0.15 – 0.61	1.18	.243
sex * task version	0.16	0.17	-0.50 – 0.18	-0.95	.676
age * sex	-0.15	0.35	-0.55 – 0.84	0.42	.346
fatigue * motivation * task version	0.14	0.21	-0.28 – 0.56	0.68	.501
age * sex * task version	0.12	0.18	-0.23 – 0.47	0.67	.503
Random effects					
σ^2	6.39	2.53			
Random intercept	9.66	3.12			
ICC	0.60				
Marginal R^2 / conditional R^2	0.03 / 0.61				

Figure S2

Percentage correct for how often each item was answered correctly in the original version.

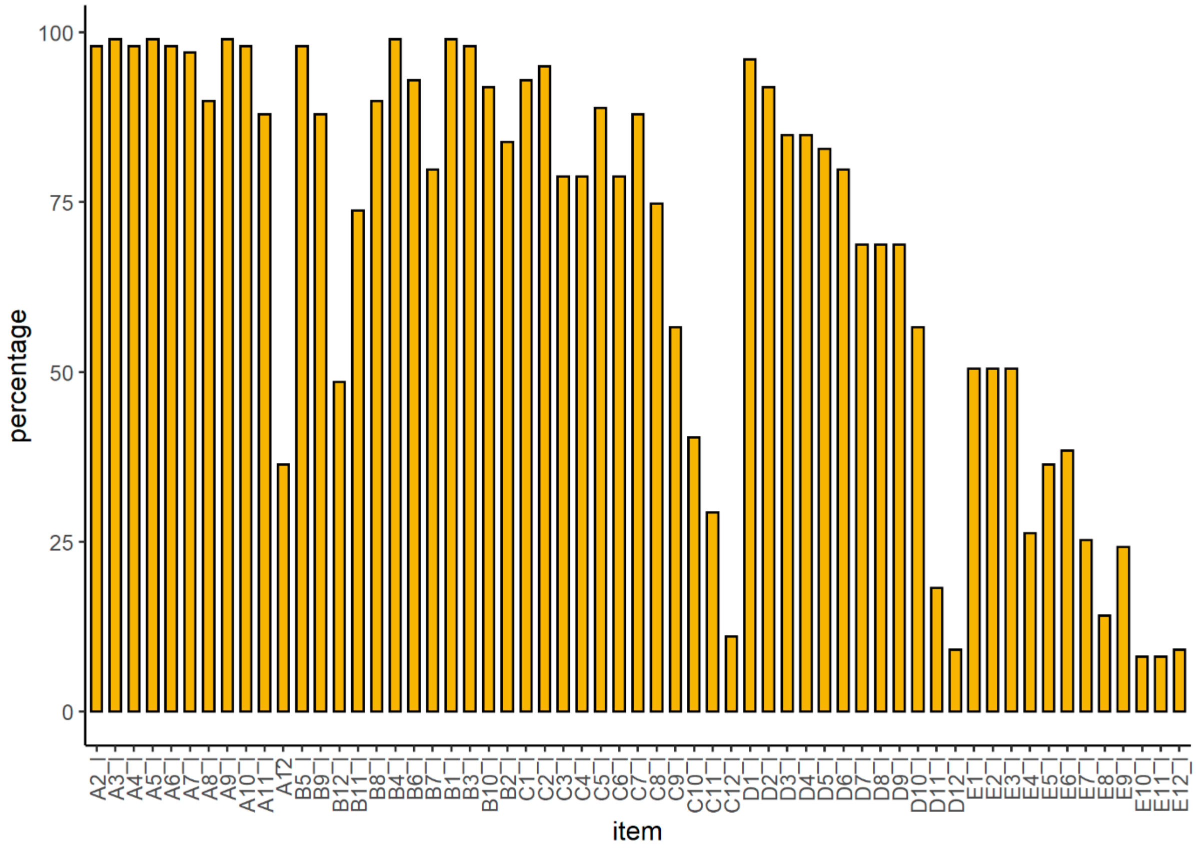
percentage
100
75
50
25
0
A2
A3
A4
A5
A6
A7
A8
A9
A10
A11
A12
B5
B9
B12
B11
B8
B4
B6
B7
B1
B3
B10
B2
C1
C2
C3
C4
C5
C6
C7
C8
C9
C10
C11
C12
D1
D2
D3
D4
D5
D6
D7
D8
D9
D10
D11
D12
E1
E2
E3
E4
E5
E6
E7
E8
E9
E10
E11
E12
item

Results exploratory logistic regression models

For the mixed effects logistic regression models, the assumption of linearity was met as there were no continuous predictors and as there is only one dichotomous predictor (i.e. version). Note that we introduced a random intercept of version, therefore accounting for potential dependency in the data due to the fact that each participant completed both versions (see Table S6 for all results). To account for multiple testing, we only considered Bonferroni corrected p-values significant when they were smaller than 0.05 / 16 = 0.003.

Table S6

Results from a series of linear mixed logistic regressions testing the effect of version on performance per item.

			Item A6			
Predictor	*b*	*SE*	*OR*	*OR 95% CI*	*z*	*p*
intercept	11.16	1.95	*NA*	*NA*	3.79	**<.001**
task version	0.00	1.11	1.00	0.11 – 8.85	0.00	.999
Other						
ICC	0.98					
Marginal R^2 / conditional R^2	0.00 / 0.98					
			Item B10			
Predictor	*b*	*SE*	*OR*	*OR 95% CI*	*z*	*p*
intercept	2.58	0.28	*NA*	*NA*	9.22	**<.001**
task version	-0.15	0.28	0.86	0.49 – 1.49	-0.53	.594
Other						
ICC	*NE*					
Marginal R^2 / conditional R^2	*NE*					
			Item B12			
Predictor	*b*	*SE*	*OR*	*OR 95% CI*	*z*	*p*
intercept	0.07	0.20	*NA*	*NA*	0.32	.746
task version	-0.15	0.16	0.86	0.63 – 1.19	-0.89	.372
Other						
ICC	0.30					
Marginal R^2 / conditional R^2	0.01 / 0.30					
			Item C11			
Predictor	*b*	*SE*	*OR*	*OR 95% CI*	*z*	*p*
intercept	-1.47	0.44	*NA*	*NA*	-2.28	**<.001**
task version	-0.13	0.20	0.88	0.59 – 1.31	-0.65	.518
Other						
ICC	0.59					
Marginal R^2 / conditional R^2	0.00 / 0.59					
			Item D1			
Predictor	*b*	*SE*	*OR*	*OR 95% CI*	*z*	*p*
intercept	23.06	4.71	*NA*	*NA*	4.90	**<.001**
task version	-8.15	3.59	0.00	0.00 – 0.33	-2.27	.023
Other						

ICC	1.00					
Marginal R^2 / conditional R^2	0.01 / 0.99					
			Item D2			
Predictor	*b*	*SE*	*OR*	*OR 95% CI*	*z*	*p*
intercept	8.69	1.75	*NA*	*NA*	4.95	**<.001**
task version	-1.12	0.65	0.33	0.09 – 1.17	-1.72	.086
Other						
ICC	0.95					
Marginal R^2 / conditional R^2	0.02 / 0.95					
			Item D4			
Predictor	*b*	*SE*	*OR*	*OR 95% CI*	*z*	*p*
intercept	7.44	1.47	*NA*	*NA*	5.08	**<.001**
task version	-0.89	0.46	0.41	0.17 – 1.02	-1.92	.055
Other						
ICC	0.94					
Marginal R^2 / conditional R^2	0.01 / 0.94					
			Item D6			
Predictor	*b*	*SE*	*OR*	*OR 95% CI*	*z*	*p*
intercept	1.81	0.36	*NA*	*NA*	5.04	**<.001**
task version	-0.19	0.20	0.83	0.56 – 1.22	-0.94	.346
Other						
ICC	0.20					
Marginal R^2 / conditional R^2	0.01 / 0.21					
			Item D9			
Predictor	*b*	*SE*	*OR*	*OR 95% CI*	*z*	*p*
intercept	1.09	0.26	*NA*	*NA*	4.16	**<.001**
task version	-0.08	0.17	0.92	0.65 – 1.29	-0.48	.634
Other						
ICC	0.27					
Marginal R^2 / conditional R^2	0.00 / 0.27					
			Item D10			
Predictor	*b*	*SE*	*OR*	*OR 95% CI*	*z*	*p*
intercept	0.14	0.17	*NA*	*NA*	0.81	.416
task version	0.15	0.15	1.16	0.86 – 1.57	0.00	.323
Other						
ICC	0.14					
Marginal R^2 / conditional R^2	0.01 / 0.15					
			Item E2			
Predictor	*b*	*SE*	*OR*	*OR 95% CI*	*z*	*p*
intercept	-0.10	0.22	*NA*	*NA*	-0.45	.655
task version	0.13	0.17	1.14	0.82 – 1.59	0.76	.446
Other						
ICC	0.38					
Marginal R^2 / conditional R^2	0.00 / 0.38					

			Item E3			
Predictor	*b*	*SE*	*OR*	*OR 95% CI*	*z*	*p*
intercept	0.21	0.25	*NA*	*NA*	0.82	.412
task version	-0.19	0.32	0.83	0.44 – 1.56	-0.58	.561
Other						
ICC	0.25					
Marginal R^2 / conditional R^2	0.00 / 0.25					
			Item E4			
Predictor	*b*	*SE*	*OR*	*OR 95% CI*	*z*	*p*
intercept	-1.86	0.46	*NA*	*NA*	-4.03	**<.001**
task version	0.23	0.21	1.26	0.84 – 1.88	1.10	.270
Other						
ICC	0.48					
Marginal R^2 / conditional R^2	0.01 / 0.49					
			Item E5			
Predictor	*b*	*SE*	*OR*	*OR 95% CI*	*z*	*p*
intercept	-0.88	0.30	*NA*	*NA*	-2.89	**.004**
task version	-0.02	0.19	0.98	0.68 – 1.41	-0.11	.915
Other						
ICC	0.49					
Marginal R^2 / conditional R^2	0.00 / 0.49					
			Item E6			
Predictor	*b*	*SE*	*OR*	*OR 95% CI*	*z*	*p*
intercept	-0.75	0.23	*NA*	*NA*	-3.21	**.001**
task version	0.13	0.17	1.14	0.82 – 1.59	0.76	.447
Other						
ICC	0.30					
Marginal R^2 / conditional R^2	0.00 / 0.30					

Table S7

Results from linear model testing whether fatigue and motivation differences predict performance differences.

			Performance difference		
Predictors	*b*	*SE*	95% CI	*t*	*p*
Intercept	-4.09	1.03	-6.14 – -2.05	-3.97	**<.001**
fatigue difference	-0.28	1.08	-2.43 – 1.86	-0.26	.793
motivation difference	-0.51	1.08	-2.67 – 1.64	-0.47	.638
Other					
R^2 / adjusted R^2	0.003 / -0.018				
Degrees of freedom	96				

References

(Pfadt et al. 2022) Pfadt, Julius M., Bergh, Don Van Den, Sijtsma, Klaas, and Eric-Jan Wagenmakers. 2022. A tutorial on Bayesian single-test reliability analysis with JASP. *Behavior Research Methods* 1–10. https://doi.org/10.3758/s13428-021-01778-0.

(Schielzeth et al. 2020) Schielzeth, Holger, Niels J. Dingemanse, Shinichi Nakagawa, David F. Westneat, Hassen Allegue, Céline Teplitsky, Denis Réale, Ned A. Dochtermann, László Zsolt Garamszegi, and Yimen G. Araya-Ajoy. 2020. Robustness of linear mixed-effects models to violations of distributional assumptions. *Methods in Ecology and Evolution* 11: 1141–52. https://doi.org/10.1111/2041-210x.13434.
